# Limb Preference and Skill Level Dependence During the Imagery of a Whole-Body Movement: A Functional Near Infrared Spectroscopy Study

**DOI:** 10.3389/fnhum.2022.900834

**Published:** 2022-06-06

**Authors:** Selina C. Wriessnegger, Kris Unterhauser, Günther Bauernfeind

**Affiliations:** ^1^Institute of Neural Engineering, Graz University of Technology, Graz, Austria; ^2^Independent Researcher, Hannover, Germany

**Keywords:** fNIRS (functional near infrared spectroscopy), motor imagery, climbing, whole-body movement, hemodynamic response

## Abstract

In the past years motor imagery (MI) turned out to be also an innovative and effective tool for motor learning and improvement of sports performance. Whereas many studies investigating sports MI focusing on upper or lower limbs involvement, knowledge about involved neural structures during whole-body movements is still limited. In the present study we investigated brain activity of climbers during a kinesthetic motor imagery (KMI) climbing task with different difficulties by means of functional near infrared spectroscopy (fNIRS). Twenty healthy participants were split into two groups according to their climbing skill level. The aim of the current study is investigating neural correlates of a whole-body sports MI task with an additional focus on skill level dependency. Climbing experts and non-experts imagined bouldering an “easy” and “difficult” route from a first-person perspective while hemodynamic responses were recorded simultaneously. We found significant differences between the two climbing routes, easy and difficult within participants as well as between the two groups of different climbing skill levels. Overall beginners showed increased hemodynamic responses compared to experts in all defined regions of interest (ROI) supporting the claim of the neural efficiency hypothesis (NEH). Even though climbing is a complex, coordinated movement of upper and lower limbs we found a stronger activation focus of the upper limbs, especially of the dominant hand-area, while the foot area seems to be deactivated or inhibited simultaneously. Summarizing, these findings provide novel insights into brain activation during the imagery of a whole-body movement and its relation to climbing expertise.

## Introduction

Motor imagery (MI) refers to the mental visualization of body movements. In sports it is a widely used approach to evaluate and increase athletes’ physical and psychological performances (Houghton, 1991; Savoy, 1993; Collet et al., 2011; MacIntyre et al., 2018; Wright et al., 2021). In this field it is mainly used to assist the progress of learning a new movement, correcting or optimizing a movement or internalizing specific moves and strategies in team sports (Guillot et al., 2008); Guillot et al. (2008, 2012) found in their work on tennis players that MI training improves serve accuracy and regularity. Other studies using MI training as an additional training method support the previous findings regarding performance enhancement among athletes (Robin et al., 2007; Battaglia et al., 2014). In a previous fMRI study we investigated the short time exercises of different sports on following MI performance (Wriessnegger et al., 2014). We found that only ten minutes of training are enough to boost MI patterns in motor related brain regions [premotor cortex (PMC) and supplementary motor area (SMA)] but also fronto-parietal and subcortical structures. This supports previous findings that MI has beneficial effects especially in combination with motor execution when used in motor rehabilitation or motor learning processes. Even though many studies already investigated the effects of mental practice they are also controversially discussed (Feltz and Landers, 1983; Murphy, 1994; Holmes and Calmels, 2008; Agosti and Sirico, 2020; Ladda et al., 2021). In the following sections we describe some findings regarding the impact of expertise in sports MI in general and specifically some effects of motor imagery during climbing.

### Sports Motor Imagery and Neural Efficiency in Experts

For example, Schack et al. (2014) concluded a connection between mental representation structures, performance, and expertise. He also concluded that this link affects the development of movement expertise among athletes (Schack and Tenenbaum, 2004; Schack and Hackfort, 2012). Moreover, long-term training leads experts to develop a focused and more efficient organization of task-related neural networks. This assumption corresponds with observations that have been made in other fields of neuroscience and supports the so-called Neural Efficiency Hypothesis (NEH). The NEH was first proposed in a PET study by Haier et al. (1992) revealing significant Glucose Metabolic Rate (GMR) changes correlating with a score on a “Tetris”-based learning task leading to the hypothesis of a more efficient energy consumption in experts’ brains compared to novice (Haier et al., 2004). The NEH has been extensively tested in the context of cognitive tasks like memory tasks (Ruff et al., 2003; Grabner et al., 2004, 2006) or intelligence (Neubauer et al., 2002; Neubauer and Fink, 2009).

In the last years, several research outcomes have extended the NEH to cortical motor systems of “experts” (Haufler et al., 2000; Kita et al., 2001; Di Russo et al., 2005; Vickers and Williams, 2017).

In sports the NEH describes how more efficient cortical neural networks and reduced neural activity are present among high performance individuals compared to individuals with poor performance.

That is “Neural efficiency” posits that neural activity is reduced in sports experts (Loze et al., 2001; Ross et al., 2003; Baumeister et al., 2008; Babiloni et al., 2009, 2010; Costanzo et al., 2016; Guo et al., 2017; Qiu et al., 2019; Zhang et al., 2019; Yang et al., 2020). In a more recent study, Seidel-Marzi et al. (2021) and colleagues investigated task-related hemodynamic responses during slacklining by means of functional near infrared spectroscopy (fNIRS). They found that more experienced slackliners exhibit less cortical activity within the right PMC, the SMA and right primary motor cortex (M1) during slacklining execution. Their findings provide novel insights into neural processing during a whole-body execution task and its relation to expertise. While they investigated neural correlates of the execution of slacklining, to date, there is a lack of knowledge with regards to capturing brain activation during the imagery of whole-body sports, like climbing.

There is only one study by Carius et al. (2020) who investigated hemodynamic responses of 13 advanced climbers during the execution of bouldering by means of fNIRS. Beside an overall activation of almost all areas of the sensorimotor system during bouldering they also found that higher bouldering expertise is related to lower SMA activity.

Even though many studies provided evidence for a reduced neural activation in experts during different kinds of sports, there are also claims suggesting that the NEH does not fully account for the organization of motor systems in elite athletes. For example, Del Percio et al. (2008) reported in their EEG study controversial results in terms of brain activity and expert level. They conclude that neural efficiency might depend on several factors including side of the movement, hemisphere, and kind of sports.

### Motor Imagery of Climbing

There is evidence from behavioral studies that utilizing mental visualization can significantly improve the skill level of climbers (Horst, 2010; Stanković et al., 2011; Naderi et al., 2018; Watt and Morris, 2020). More concretely, MI is a useful tool to plan the physical movements and weight shifts across a route to be climbed. People who have climbed for longer, generally visualize the goal route first, then they imagine the movements necessary to accomplish that route, while looking at it, and lastly execute the movements to climb. This behavior is related to the experience climbing experts have gained over several years. Compared to that novices follow a “learning by doing” strategy where they usually think about the next movement as they climb. Most of the studies using MI of climbing concentrated more on the cognitive, visual side of imagery. That is going mentally through the route beforehand or visually imagine the perspective to plan and find a specific route (Hardy and Callow, 1999; Kiewa, 2001; Boyd and Munroe, 2003; Callow and Roberts, 2010). No studies using MI of climbing, defined as the kinesthetic imagery of a climbing task from a first-person perspective.

In the present study we used MI of climbing because it is a sport that makes use of complex, coordinated, arm and foot movements to lift one’s weight on different vertical or angled surfaces. More specific bouldering is a form of climbing performed without ropes or harness on small surfaces and it is the combination of body movements created within the kinematic and kinetic parameters which makes it clearly different from other sports. Especially its complex of coordination abilities like spatial orientation or balance involving both contraction and relaxation.

Despite some behavioral findings, relatively little is known about the underlying brain activity during the imagery of climbing *per se* and with an additional focus on expertise. In this study we investigated the following two research questions: First, which specific hemodynamic activation patterns elicit during the climbing task itself and where are the foci of activation. In view of the characteristics of climbing which requires complex, coordinated hand and foot movements we hypothesize a cortical widespread hemodynamic response pattern including upper and lower limbs over all participants. Related to the cognitive nature of the task we assume concentration changes in [oxy-Hb] in motor related areas, but also in frontal regions of the cortex.

Second, is there a difference between hemodynamic activation patterns regarding the two climbing routes? We hypothesize an increase in [oxy-Hb] concentration changes for the difficult route in beginners.

Third, do the hemodynamic activation patterns during the imagery of climbing differ between experts and non-experts? We hypothesize that mean hemodynamic alterations differ between groups of different skill levels supporting the NEH. More concretely we will expect higher [oxy-Hb] concentration changes in the frontoparietal network of beginners compared to experts.

## Materials and Methods

### Participants

Twenty-two healthy right-handed participants aged 23–41 years (mean = 30.14, SD = ± 7.2) took part in this study. All participants were informed in written form about the aim of the study, the measurement technique, and signed informed consent prior to the experiment. Handedness assessment was calculated for each subject through the Hand Dominance Test (HDT; Steingrüber and Lienert, 1971). This paper-and-pencil test consists of three different subtasks (tracing lines, dotting circles, tapping on squares) each to be performed with maximal speed and precision for 15 s. Hand dominance can theoretically vary from –100 (extreme left-handedness) to +100 (extreme right-handedness). A total HDT score for the laterality coefficients (R-L)/(R + L) × 100 was calculated for each volunteer. Beginners received a mean HDT score of 98.1 (SD:7.3) and experts 97.2 (SD: 4.8), indicating a clear right-handedness of persons in both groups.

The study was approved by the local ethics committee (Medical University of Graz) and was in accordance with the ethical standards of the Declaration of Helsinki. The participants were divided into two groups according to their climbing skill level, namely novice and experts and balanced to gender and age. The climbing skill level of the participants was investigated by the use of a questionnaire, focusing on how many times per week and since when the respective participant performed bouldering. The mean weekly training hours in the expert group was 7.4 (SD: 1.35; Minimum: 6 and Maximum 10 h/week) and in the beginner’s group 2 (SD 0.75; Minimum: 1.5 and Maximum: 3 h/week). Furthermore, the mean years of climbing experience in the group of experts was 5.2 (SD:1.25) years and in the group of beginners 0.8 (SD: 0.33).

Accordingly participants were assigned to the beginner and experts group. Overall experts stated climbing about 3 times a week, for 2 h and at least 5 years. Novices started to climb some months ago with a minimum frequency of 1 climbing sessions per week with an average duration of 1.5 h.

### Tasks and Experimental Procedure

Before the start of the experiment a measurement protocol, including demographic features was filled out together with the participants. Additionally, they had to answer some questions regarding their climbing expertise, concretely they had to indicate since when they are climbing, how often per week and how long their typical climbing sessions are. After that the HDT was performed to assess the handedness of the persons. After the collection of participant specific variables, the subjects were introduced to the task of the experiment. They were asked to imagine two different boulder problems from a first-person perspective, as shown on a screen. We used two boulder problems of different skill levels: 5a and 6a according to the Fontainebleau grading system. This grading system is the most widely used in Europe. 1A–1C is reserved for slabs that can be climbed without the use of hands. 2A–2C is generally yellow or green, and signify good holds. 3A–3C (Orange), are designated where technique is needed, 4A–4C (blue), is for problems that require either specific arm or fingertip strength, 5A–5C (red), is given for problems that have a combination of fingertip and arm strength and 6A–6C (black or white), are very hard and sometimes desperate routes (Draper et al., 2015). The levels were explicitly defined for this study by a climbing professional and staff member of a local climbing hall.

Before starting the experimental procedure, participants were shown pictures of the two boulder problems on a DIN A4 paper. These included indications for hand and foot placements at the starting holds and at the top. Participants were asked if it was possible for them to imagine climbing toward the top including hand and foot movements to ensure the participants fluent MI performance during the task and hence, enable a useful contribution to meet this study’s purpose. Additionally, subjects were indicated to grasp two provided climbing holds for 30 s to increase their kinesthetic imagery ability in the following tasks.

**FIGURE 1:**
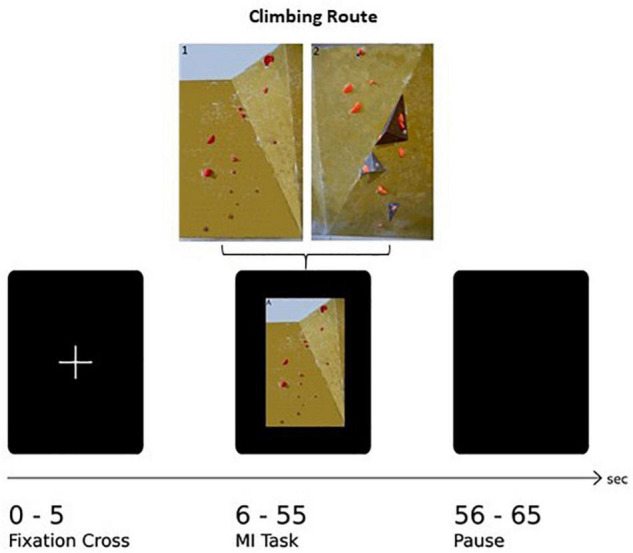
Illustration of one experimental trial: After the presentation of a fixation cross (5 s) the image of the climbing route (exemplarily Route 1: easy) is presented (50 s) followed by a rest period (10 s).

The paradigm of this task consists of 30 trials in total, with 15 trials per route. The trial sequence was shown in a pseudo-randomized order, guaranteeing the same route occurring not more than twice subsequently. One trial consisted of three different sequences: The first 5 s, a white fixation cross on black screen was shown followed by the appearance of a picture of one of the two boulder problems for 50 s. To bring subjects’ cortical activity back to baseline, a black screen was presented at the end of one trial. One whole trial therefore lasts 65 s leading to a total of 32.5 min for the whole paradigm. In addition, at the very beginning and the very end a short information indicated the start and the end of the experiment, respectively. An example of a whole paradigm sequence is shown in Figure 1. The paradigm was presented with a custom script in MATLAB (The Math-works Inc., Natick, MA, United States) on a vertically oriented 27-inch monitor screen and synchronized with the fNIRS data acquisition system. Furthermore, the whole experiment was video monitored so that the experimenter can intervene if actual movements occur.

### Measurement Procedure and Data Acquisition

In order to enable careful correction of artifacts induced by various bio signals on the recorded fNIRS data, physiological signals such as electrocardiogram (ECG) and respiration (RP) were recorded for each participant. The ECG was recorded bipolar through electrodes placed on the thorax at positions V2 and V5 and a reference electrode placed above the innominate bone. The RP was recorded with a respiratory sensor (Respiratory Effort Sensor, Pro-Tech Services, United States). For recording the ECG and RP, a bio signal amplifier (g.USBamp Guger Technologies, Austria) with a sampling rate of 256 Hz was used (Wriessnegger et al., 2018). Subjects were asked to take place in an armchair located in front of a computer screen in a dimmed room. After measuring the head circumference, probes were placed on an appropriate cap (54, 56, and 58 cm) according to the international 10–20 system. In addition to the closing of the straps below the chin, the cap was fixed diagonally on a mounted chest strap to guarantee the caps stability during the experiment. The hemodynamic responses were recorded with the continuous-wave (CW) based fNIRS system “NIRScout – NIRS Imaging System” (NIRx Medical Technologies LLC, Glen Head, United States) using the modified Beer-Lambert law. Since CW systems cannot measure the tissue optical pathlength (for a review see Ferrari and Quaresima, 2012) the scale unit of the system is the molar concentration multiplied by the unknown pathlength (mM mm). The near-infrared light was emitted with 760 and 850 nm wavelengths and data were recorded with a sampling frequency of 3.91 Hz. The system consisted of 16 sources and 22 detectors arranged in an order around detector 12 at position Cz resulting in 61 channels distributed among the frontal, central and parietal cortex (Wriessnegger et al., 2018) with an inter-optode distance of 3 cm.

**FIGURE 2:**
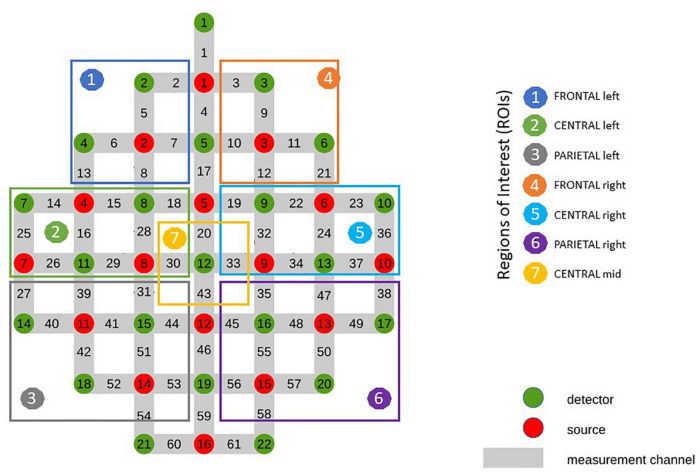
Optode Montage and Regions of Interest (ROI).

For the subsequent analysis related channels were averaged resulting in 7 different ROIs (Figure 2), covering frontal (RO11: average of channels 2, 5, 6, 7, 13, 8/ROI4: average of channels: 3, 9, 10, 11, 12, 21), central (ROI2: average of channels 14, 15, 18, 25, 16, 28, 26, 29/ROI 5: average of channels 19, 22, 23, 32, 24, 36, 33, 34, 37) and parietal (ROI3: average of channels 27, 39, 31, 40, 41, 44, 42, 51, 52, 53/ROI6: average of channels 35, 47, 38, 45, 48, 49, 55, 50, 56, 57, 58) areas of both hemispheres and mid central (ROI7: average of channels 20, 30, 33, 43) were used. The classification of the ROIs was based on a prior bachelor thesis at our institute dealing with projections of topographical data based on skull landmarks into a 3D reference frame [MNI-space, Montreal Neurological (Singh et al., 2005)]. For each fNIRS channel position, a set of MNI coordinates (x, y, and z) was calculated together with an error estimate (SD). The centers of the circle regions represent the locations of the most likely MNI coordinates for the fNIRS channel projected on the cortical surface. In the present study ROI1 and ROI4 were defined by channels overlaying left/right frontal areas, comprising Brodmann areas (BA) 9, 10. ROI2 and ROI5 was defined by channels overlaying central, motor-related areas, comprising BA 6 and BA 8 and ROI3 and ROI6 was defined by channels overlaying parietal, somatosensory regions, comprising BA 3, 5 and BA 40. ROI7 was defined as a distinct central position covering the foot area of the motor cortex, comprising BA 1, 4, and 5.

The calibration of the sources and detectors as well as the signal acquisition was performed using the software “NIRStar 14-1” (NIRx Medical Technologies LLC, Glen Head, United States) in combination with the paradigm implemented into a custom written MATLAB (The Math- works Inc., Natick, MA, United States) script performed on a Lenovo ThinkPad R500 (Lenovo Group Ltd., Peking, China) connected to the biosignal amplifier.

### Data Analysis

#### Data Pre-processing

At the very beginning of the pre-processing pipeline, a visual inspection of the raw fNIRS data regarding the removal of possible noisy channels for further data processing steps was performed. Thereby, “bad” signals were compared with the “bad” channels manually notated while calibrating before the start of the experiment. Additionally, data was visualized concerning motion artifacts. For subsequent processing steps custom-written MATLAB scripts were used. Physiological induced artifacts, namely “Mayer Waves,” heart- and respiration rate were corrected according to their origins frequency band, since they interfere with the fNIRS signal (de Boer et al., 1986; Bauernfeind et al., 2014). This was realized by using transfer function (TF) models which allow to reduce the interference of the various signals (Bauernfeind et al., 2014). Heart rate was defined as a source of “Mayer Waves” and their induced artifacts were corrected by using a band-pass filter with bandwidth 0.07–0.13 Hz. Respiration and the pulse peak of the heart rate were band-pass filtered between 0.2–0.4 Hz and 0.5–1.5 Hz, respectively (Bauernfeind et al., 2014). Additionally, a notch filter and baseline correction with band-stop 0.005 and 0.1 Hz were applied. For further analyses the task-related mean concentration of oxygenated hemoglobin [Oxy-Hb] and deoxygenated hemoglobin [Deoxy-Hb] was estimated and averaged for each channel (Wriessnegger et al., 2018). The measured area was divided into 7 regions of interest (ROIs) covering the left and right hemisphere respectively: ROI1 and ROI4 (frontal), ROI2 and ROI5 (central) and ROI3 and ROI6 (parietal) and the central ROI 7 (covering the foot area). For analyzing the fNIRS data the open source NICA toolbox (Raggam et al., 2020) was used.

#### Statistical Analysis

For investigating the data regarding possible effects and interactions between the different conditions we ran a 2 × 7 mixed design repeated measures ANOVA on both [Oxy-Hb] and [Deoxy-Hb] data. The within-factors were the type of climbing route (Route 1: easy; Route 2: difficult) and 7 different ROIs [left hemisphere: frontal (ROI1), central (ROI2), and parietal (ROI3) right hemisphere: frontal (ROI4), central (ROI5) and parietal (ROI6) and mid central (ROI7)]. The factor group according to the climbing level (Beginners and Experts) was used as a between-subject factor. Additionally, a paired-samples *t*-test was performed comparing each ROI and climbing routes in both groups – beginners and experts. A Tukey correction was used to control for multiple comparisons in the *post hoc* tests.

For statistical analysis the time period from 15 to 30 s was considered, since according to our experimental design and previous work the estimated concentrations of [Oxy-Hb] and [Deoxy-Hb] were assumed to be most distinct during this period. This was also inspected visually on data of randomly chosen subjects of the experiment. Furthermore, a Pearson Correlation was calculated between [Oxy-Hb] and [Deoxy-Hb] concentration changes and weekly climbing performance in hours separately. All statistical analyses were performed with the open source toolbox “jamovi” (The jamovi project (2021) *jamovi* (Version 1.6) [Computer Software]. Retrieved from https://www.jamovi.org.

## Results

### Topographical Representation of the Averaged Concentration Changes of [Oxy-Hb] and [Deoxy-Hb]

**FIGURE 3:**
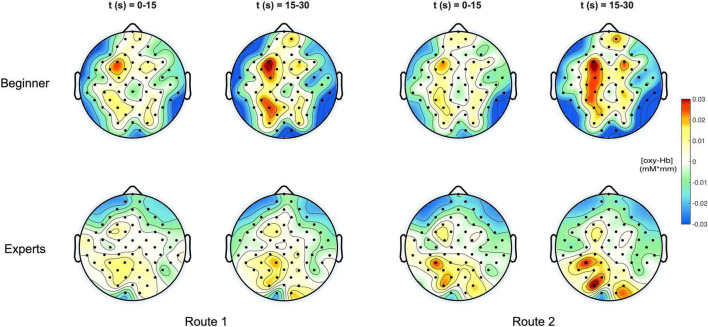
**(Top)** Topoplots of the mean oxy-Hb concentration changes for beginners during MI of both routes (left side: Route 1 = easy; right side: Route 2 = difficult) and two time windows (0–15 s and 15–30 s). **(Bottom)** Topoplots of the mean oxy-Hb concentration changes for experts during MI of both routes (left side: Route 1 = easy; right side: Route 2 = difficult) and two time windows (0–15 s and 15–30 s).

Figure 3 shows a topographical representation of the estimated concentration changes of [Oxy-Hb] averaged among the subjects for both groups “Beginners” and “Experts” respectively for the two different climbing routes, easy and difficult. Hereby, the averaged concentration values are plotted at their corresponding spatial positions (Pfurtscheller et al., 2010).

For both groups, beginners and experts, two time intervals were presented, showing the course of oxygenation from the beginning phase (0–15 s) to the end phase (15–30 s) of the task.

**FIGURE 4:**
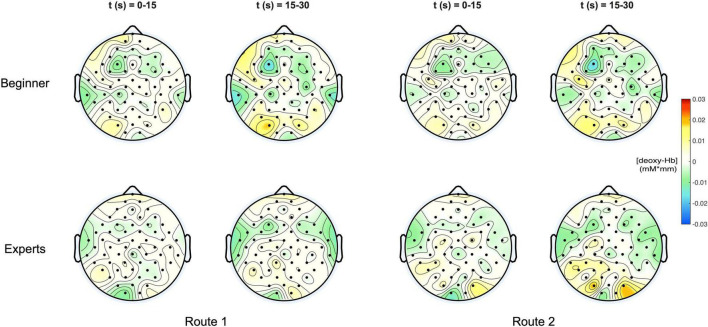
**(Top)** Topoplots of the mean deoxy-Hb concentration changes for beginners during MI of both routes (left side: Route 1 = easy; right side: Route 2 = difficult) and two time windows (0–15 s and 15–30 s). **(Bottom)** Topoplots of the mean deoxy-Hb concentration changes for experts during MI of both routes (left side: Route 1 = easy; right side: Route 2 = difficult) and two time windows (0–15 s and 15–30 s).

In Figure 4 the topographic distribution of [deoxy] Hb concentration changes are plotted for the same conditions like in Figure 3. Since neural activation during motor tasks is primarily reflected in [oxy] Hb concentration changes (Miyai et al., 2001, 2002; Strangman et al., 2002), no significant effects were observed for [deoxy] Hb.

### Statistical Results

The mixed ANOVA for repeated measures was performed on concentration changes of both [Oxy-Hb] and [Deoxy-Hb]. However, the statistics only showed significance for concentration changes of [Oxy-Hb], which are exclusively reported here. For a correct interpretation of the statistical results a Greenhouse–Geisser correction was used if the assumption for sphericity tested by Mauchly’s test did not hold. The 2 × 7 repeated measures ANOVA on [Oxy-Hb] showed a significant main effect for the within-condition ROI [*F*(2.57,43.73) = 3.72, *p* = 0.002, η_p_^2^ = 0.18]. The second within-factor “ROUTE” also revealed significance [*F*(1, 17) = 40.59, *p* ≤ 0.001, η_p_^2^ = 0.71]. Furthermore the following interactions reached significance: ROI × ROUTE [*F*(2.79,47,48) = 9.67, *p* ≤ 0.001, η_p_^2^ = 0.36], ROI × ROUTE × GROUP [*F*(2.79,47,48) = 5.21, *p* = 0.004,ηp^2^ = 0.23)]. Additionally we found significant differences for the between factor “GROUP” [*F*(1,17) = 6.95, *p* = 0.009, η_p_^2^ = 0.24]. *Post hoc* analysis with correction for multiple comparisons (Tukey-Test) revealed significant differences between beginners and experts, especially over frontal regions for Route 1 [*t*(21) = –3.82, *p* = 0.044] and Route 2 [*t*(21) = –2.185, *p* = 0.041]. But also significant differences between hand related central cortical areas (ROI2) and the foot area (ROI7) were observed in beginners when comparing Route 1 [*t*(21) = 3.486, *p* < 0.001] and Route 2 [*t*(21) = 3.987, *p* < 0.001]. The results of the Pearson Correlation showed no significant correlation between the number of training hours per week and [oxy-Hb]/[deoxy-Hb] concentration changes at all. Table 1 shows means and standard deviations of [oxy-Hb] for beginners and experts as a function of ROI (7) × Route (2) design. Significant differences within the groups between conditions are highlighted.

**TABLE 1:** Descriptive statistics: means and standard deviations of [Oxy-Hb] concentration changes for both groups (Beginners and Experts), both Routes (1: easy and 2: difficult) for each ROI (ROI1-ROI7) are illustrated.

	Beginners (*N* = 11)				Experts (*N* = 11)			
ROIs	Route 1		Route 2		Route 1		Route 2	
	Mean	SD	Mean	SD	Mean	SD	Mean	SD
ROI1	–0.0029	0.0116	–0.0031	0.0152	–0.0082	0.0064	–0.0095	0.0153
ROI2	0.0149	0.0172	0.0170	0.0146	0.0024	0.0077	0.0024	0.0073
ROI3	0.0096	0.0104	0.0176	0.0111	0.0076	0.0102	0.0070	0.0115
ROI4	–0.0047	0.0216	0.0026	0.0239	0.0040	0.0149	–0.0002	0.0191
ROI5	0.0050	0.0108	–0.0003	0.0147	0.0064	0.0085	0.0103	0.0235
ROI6	–0.0077	0.0239	–0.0126	0.0209	–0.0033	0.0124	–0.0046	0.0118
ROI7	–0.0152	0.0087	0.0052	0.0070	–0.0161	0.0087	0.0044	0.0071

*Significant differences (α = 0.05) between conditions within the groups are highlighted in gray.*

**FIGURE 5:**
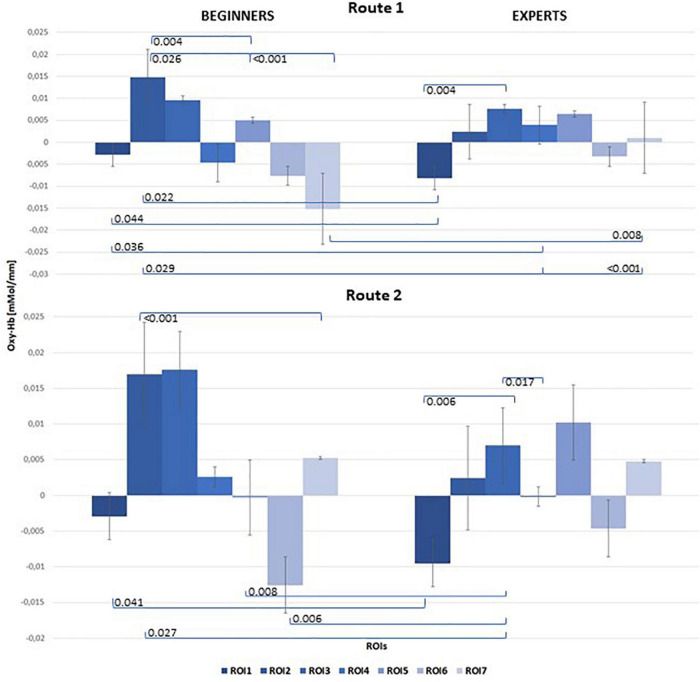
Bar chart of mean [oxy-Hb] concentration changes for both groups (left: Beginners; right: Experts), all 7 ROIs and both Routes: top level: Route 1 = easy; bottom level: Route 2 = difficult. Significant results of *post hoc* comparisons between beginners and experts were indicated with Tukey-test corrected *p*-values (α = 0.05).

Figure 5 illustrates the mean concentration changes of [oxy-Hb] with all significant results between beginners and experts for both routes.

## Discussion

In the present study, we investigated brain activity on the basis of hemodynamic response alterations during the kinesthetic MI of climbing between two groups of different climbing skill levels. By comparing experts and novices during MI of climbing, we also contributed to an unexplored topic, as neural processing of such whole-body imagery task was not targeted in previous studies so far. In our study participants with different skill levels imagined climbing up two different bouldering routes (easy/difficult) in order to reveal task-related hemodynamic activation patterns of MI based on expertise. Based on the literature (Strangman et al., 2002; Obrig and Villringer, 2003; Plichta et al., 2007; Bauernfeind et al., 2018) and our previous experience (Wriessnegger et al., 2008, 2012, 2017, 2018) we focused on [oxy-Hb] concentration changes as the relevant variable associated with brain activity. The analysis for [deoxy-Hb] hemodynamic changes did not show any significance which might be due to the lower signal-to-noise ratio (Huppert et al., 2006) and its lesser contribution to cortical activation (Gagnon et al., 2012). Furthermore Hoshi et al. (2001) suggested that, in activation studies with near-infrared spectroscopy, [Oxy-Hb] is the most sensitive indicator of changes in cerebral blood flow (CBF) and consequently of changes in neural activation patterns.

### Activation Patterns and Neural Efficiency

In the group of experts, the overall activation was lower for both routes with higher [oxy-Hb] concentration changes over parietal regions. In beginners an overall increase of the range of [Oxy-Hb] can be observed on both climbing routes.

For both routes an increased activation is found in the left hemisphere over central and parietal ROI. According these results our second hypothesis regarding expected differences in route difficulty was rejected.

Overall significantly lower activation patterns were observed in the group of experts supporting the claim that expertise impacts neural activity as evidenced by a significant reduction in hemodynamic alterations among experts. Even though this is in line with the assumptions of the NEH and further studies (Ross et al., 2003; Wei and Luo, 2010; Guo et al., 2017; Seidel-Marzi et al., 2021) it should be also mentioned that others reported controversial effects (Li and Smith, 2021). Therefore, it is important to define the NEH as a dynamic and situational concept that strongly depends on several factors including movement characteristics (e.g., side, effectors, strength), hemisphere, and of course athletes’ traits and type of sports. Future neuroimaging studies are necessary to investigate such factors which might influence the NEH in a more standardized way.

### Activation Patterns and Lateralization Effects

Stronger left sided activation patterns were observed in parietal and central ROI in both groups. For the “difficult” climbing condition a similar cortical distribution of hemodynamic changes is observed with additional activity over the left primary motor cortex. In both tasks, a distinct activation of the left hemisphere, including parts of the prefrontal cortex, the supplementary motor cortex, the PMC and the somatosensory motor cortex, was observed. These findings correspond with the results from previous studies which reveal a link between kinesthetic MI and increased activity in the left inferior parietal lobule and the left somatosensory cortex (Ruby and Decety, 2003; Stinear et al., 2006) but even though the lateralization effect was quite surprising in our study since climbing involves whole-body movements and especially the involvement of both upper limbs.

It seems that handedness plays an important role and that the dominant hand-system, all participants were right-handed, takes over control in performing complex whole-body motor tasks. This assumption is supported by the theory of Mutha et al. (2013) who suggested that motor lateralization is the result of a specialization of each arm-hemisphere system for distinct and complementary motor control mechanisms (Mutha et al., 2012, 2013; Sainburg, 2014). They propose that the dominant arm-hemisphere (for right-handers the left hemisphere) is more specialized for dynamic features of movements like its direction or trajectory shape (Bagesteiro and Sainburg, 2002; Schaefer et al., 2009; Mutha et al., 2011). The basis for such a control mechanism is prediction and anticipation of dynamic properties of arm control in the environment. On the other hand the non-dominant hemisphere is more specialized to achieve stable postures by keeping the balance (Duff and Sainburg, 2007; Wang and Sainburg, 2007). Following this the non-dominant arm often shows higher precision and control in reaching a specific spatial position. Following this Dynamic Dominance Model (Sainburg, 2014) the right hemisphere controls processes that can minimize potential errors when faced with unexpected events, and can achieve accurate steady-state positions. In contrast to other researchers they suggest that both systems coexist in the brain and are not in favor of one or the other in controlling arm movements. This might be an explanation for the stronger activation patterns in the dominant hemisphere of the participants in our study. It seems that during the imagery of climbing dynamic features of the movement, like direction, are more important than spatial positions and balance.

### Limb Specific Activation Patterns

In view of the characteristics of the climbing task we hypothesized cortical widespread hemodynamic response patterns including upper and lower limbs over all participants. Indeed, we observed stronger activation patterns only in the left hemisphere over the cortical hand representation areas which is in line with previous EEG studies (Pfurtscheller and Neuper, 1997; Neuper et al., 2006; Nicolas-Alonso and Gomez-Gil, 2012; Stefano Filho et al., 2020). But more interestingly beside the increased activity over hand related cortical areas we observed a simultaneous decrease of activation over temporal parts of the cortex and the foot area. These results suggest that specific brain areas might be deactivated/inhibited or less involved in performing the MI task (Gusnard et al., 2001; Raichle and Snyder, 2007) which might be comparable with a neural phenomenon called “focal ERD (event-related desynchronization)/surround ERS (event-related synchronization).” This effect describes the observation that desynchronization of alpha (mu) rhythm occurs not in isolation but can be accompanied by an increase in synchronization in neighboring cortical areas that correspond to the same or to another modality (Lindig-León et al., 2020; Pfurtscheller et al., 1999; Suffczynski et al., 2001). Basically, MI of hand movements activates neural networks in the cortical hand representation area, manifested as blocking or desynchronization of the hand area mu rhythm (Neuper et al., 2006) by simultaneously inducing a synchronization (ERS) in neighboring areas, like the foot area. Such antagonistic behavior was also described in an fNIRS study by Pfurtscheller et al. (2010). They found a significant simultaneous [oxy-Hb] increase in the dorsolateral prefrontal cortex (DLPFC) accompanied by a [oxy-Hb] decrease in the anterior prefrontal cortex (APFC) during simple mental arithmetic tasks. Other studies showed that such inhibition (or “idling” state) of the hand area networks can occur when the motor attention is directed to the foot or tongue modalities, and attention is withdrawn from the hand.

Here we found stronger activation of the hand area compared to the foot area even though bouldering requires the combination of complex multi-limb activities. It seems that during the imagination of climbing, the upper limbs have a stronger focus leading to higher [oxy-Hb] concentration changes, while the foot area seems to be deactivated or inhibited simultaneously.

An alternative explanation could be the anatomical localization of the foot area, between the two hemispheres, which is much harder to capture compared to the hand area. In comparison to right- or left-hand MI, activated areas are larger and located on the surface of the brain, whereas for foot MI the corresponding areas are located in interior sections of the medial longitudinal fissure which are not fully penetrated by the near infrared light (Kaiser et al., 2014; Li et al., 2021). Thus, the lack of different patterns might also be due to the limited penetration depth (about 25 mm, Okada et al., 1997) of the fNIRS method. Furthermore, previous studies reported that it is nearly impossible making a distinction between left and right foot imagery, since the related cortical areas are too close to generate identifiable signals (Stippich et al., 2002; Graimann et al., 2009).

### Study Limitations

Despite the strengths of the current study, which include novel findings in hemodynamic alterations during climbing, there are some limitations that should be noted. First, the small sample size within the groups could be a lack of the study. Nevertheless, our results could be very interesting for designing future neuroimaging studies investigating MI in whole-body sports. Second, the use of a questionnaire (e.g., “Vividness of Movement Imagery Questionnaire”) capturing MI ability of participants could be an additional beneficial source of information not considered in the current study but will be integrated in our future studies. Third, the absence of EMG control during the imagery phases might also be a limitation and should be implemented in future studies focusing on MI.

## Conclusion

We investigated the brain activity by means of fNIRS during the imagery of climbing in persons with different climbing skill levels. Stronger hemodynamic responses reflected by significantly higher [oxy-Hb] concentration changes were found in beginners compared to expert climbers supporting the NEH hypothesis. Furthermore, an antagonistic behavior of activation could be observed in relation to the region of interest. That is, the kinesthetic imagery of climbing leads to a stronger activation of the hand area by simultaneously inhibiting the foot area. Summarizing our findings contribute to a relatively unexplored field, as brain functioning during the imagery of climbing was not targeted in studies so far. Furthermore, it might have implications beyond improving climbing skills of athletes, as MI is in general crucial in the context of prevention and rehabilitation, especially in neurodegenerative motor diseases or stroke.

## Data Availability Statement

The raw data supporting the conclusions of this article will be made available by the authors, without undue reservation.

## Ethics Statement

The studies involving human participants were reviewed and approved by the Medical University of Graz. The participants provided their written informed consent to participate in this study.

## Author Contributions

KU and SW conceived and designed the experiments, analyzed the data, and writing-original draft. KU performed the experiments. SW and GB supervised and writing – review and editing. All authors contributed to the article and approved the submitted version.

## Conflict of Interest

The authors declare that the research was conducted in the absence of any commercial or financial relationships that could be construed as a potential conflict of interest.

## Publisher’s Note

All claims expressed in this article are solely those of the authors and do not necessarily represent those of their affiliated organizations, or those of the publisher, the editors and the reviewers. Any product that may be evaluated in this article, or claim that may be made by its manufacturer, is not guaranteed or endorsed by the publisher.
